# Recording Synaptic Transmission from Auditory Mixed Synapses on the Mauthner Cells of Developing Zebrafish

**DOI:** 10.1523/ENEURO.0021-22.2022

**Published:** 2022-06-20

**Authors:** Fabio A. Echeverry, Sundas Ijaz, Alberto E. Pereda

**Affiliations:** Dominick P. Purpura Department of Neuroscience, Albert Einstein College of Medicine, Bronx, NY 10461

**Keywords:** auditory afferent, connexin, electrical synapse, gap junction, glutamate, mixed transmission

## Abstract

The Mauthner cells are a pair of large reticulospinal neurons that organize sensory-evoked tail flip responses in fishes. An identifiable group of auditory “mixed” (electrical and chemical) synaptic contacts known as “Large Myelinated Club endings” on these cells have provided a valuable model for the study of synaptic transmission in the vertebrate brain. While most of studies were performed in adult fish, we describe here methods that make possible recording synaptic transmission from these contacts in developing zebrafish, a genetically tractable vertebrate species which is uniquely amenable for combining synaptic physiology with live imaging and behavioral analysis.

## Significance Statement

An identifiable type of “mixed” synaptic contacts on the fish Mauthner cells provide a valuable model for the study of synaptic transmission in the vertebrate brain. We describe here methods to record synaptic potentials from these contacts in developing zebrafish. The approach makes possible to explore the molecular and functional properties of these model mixed synapses in a genetically tractable species at which synaptic mechanisms and their circuit functions can be more easily examined.

## Introduction

The Mauthner cells (M-cells) are a pair of unusually large reticulospinal neurons that, as a result of their sensory inputs and spinal connectivity, organize tail-flip escape responses in fish (Korn and Faber, 2005; Faber and Pereda, 2011). Because of their larger size and experimental accessibility and identifiable inputs, M-cells have also historically provided a window for investigations of synaptic mechanisms (Korn and Faber, 2005; Pereda and Faber, 2011). One of these inputs is a special class of auditory afferents originated in the fish sacculus that each terminates as a single, large, synaptic contact on the lateral dendrite of the M-cell (Bartelmez, 1915). These auditory contacts, known as “Large Myelinated Club endings” or “Club endings” (Bartelmez, 1915; Bartelmez and Hoerr, 1933; Robertson et al., 1963; Pereda et al., 2004), are thought to provide critical information for the initiation of auditory-evoked escape responses in fish (Korn and Faber, 2005; Faber and Pereda, 2011; Pereda and Faber, 2011). Beyond their functional role, Club endings have provided, since their first description by George Bartelmez (Bartelmez, 1915) a valuable model for the investigations of synaptic communication. That is, because of their larger size and typical distal distribution on the lateral dendrite of the M-cell more easily allowed correlations of synaptic structure and function (Bartelmez and Hoerr, 1933; Bodian, 1937; Robertson, 1963; Robertson et al., 1963; Furshpan, 1964; Rash et al., 2013). Investigations on Club endings provided one of the earliest evidences for the identification of the anatomic bases of electrical transmission, thus critically contributing to the identification of the ubiquitous intercellular structures that are now recognized as “gap junctions” (GJ; Robertson, 1963; Robertson et al., 1963). Moreover, at Club endings, GJs co-exist with specializations of chemical transmission (Robertson et al., 1963; Kohno and Noguchi, 1986; Tuttle et al., 1986). Consistent with this anatomic arrangement, the activation of Club endings evokes a “mixed” synaptic potential in the M-cell, at which both electrical and chemical transmission co-exist (Furshpan, 1964; Lin and Faber, 1988a, b; Wolszon et al., 1997). Seminal electrophysiological studies in Club endings exposed for the first time the activity-dependent plastic properties of electrical transmission, whose induction mechanisms results from interactions with co-existing glutamatergic areas in the contact (Yang et al., 1990; Pereda and Faber, 1996; Pereda et al., 1998; Cachope et al., 2007). These plastic interactions between the two main modalities of synaptic transmission were later also found to occur in the mammalian brain (Kothmann et al., 2012; Mathy et al., 2014; Turecek et al., 2014). Thus, by permitting correlations of anatomic and biochemical features with their physiological properties, investigations on Club endings have provided valuable insights into synaptic mechanisms, especially those underlying electrical transmission (Pereda et al., 2004).

Because of their transparency at early developmental stages and emerging complex behaviors after just 5 d of development, larval zebrafish have been recently identified as an particularly advantageous model organism for the analysis of neural circuits (McLean and Fetcho, 2009; Portugues and Engert, 2009; Ahrens et al., 2012; Friedrich et al., 2013; Feierstein et al., 2015; Koyama et al., 2016). Amongst those circuits, the M-cell and its associated network, is amenable for correlations of the escape behavior with their cellular and molecular determinants (Burgess and Granato, 2007; Burgess et al., 2009; Wolman et al., 2015; Jain et al., 2018). Club endings were first identified in larval zebrafish with electron microscopy by Kimmel (Kimmel et al., 1981), and their number and distribution, functional properties, and molecular composition more recently characterized combining confocal imaging, electrophysiology, and genetic manipulations (Yao et al., 2014; Miller et al., 2015, 2017a; Lasseigne et al., 2021). Recent studies in zebrafish Club endings revealed an unexpected complexity of the molecular organization of electrical synapses, exposing the critical functional role of the molecular scaffold at GJs and which includes an asymmetry in the presynaptic versus postsynaptic distribution of GJ components (Miller et al., 2015; Marsh et al., 2017; Lasseigne et al., 2021). Thus, contrasting other model systems, larval zebrafish Club endings provide not only an unprecedented window for the analysis of the properties of synaptic transmission at which detailed molecular mechanisms can be more thoroughly investigated by combining advanced anatomic analysis, *in vivo* imaging and electrophysiology with genetic manipulations (Wen et al., 2010, 2013, 2016a; Lasseigne et al., 2021), but the possibility of correlating them with their functional role within a circuit that generates a stereotyped, quantifiable, behavioral response. These larval zebrafish synapses will be uniquely suitable for exploring questions regarding the development of the auditory system, the molecular machinery required for formation and function of electrical and chemical transmission, exposing developmental and functional interactions between chemical and electrical synapses, and their behavioral correlates.

**Figure 1.**
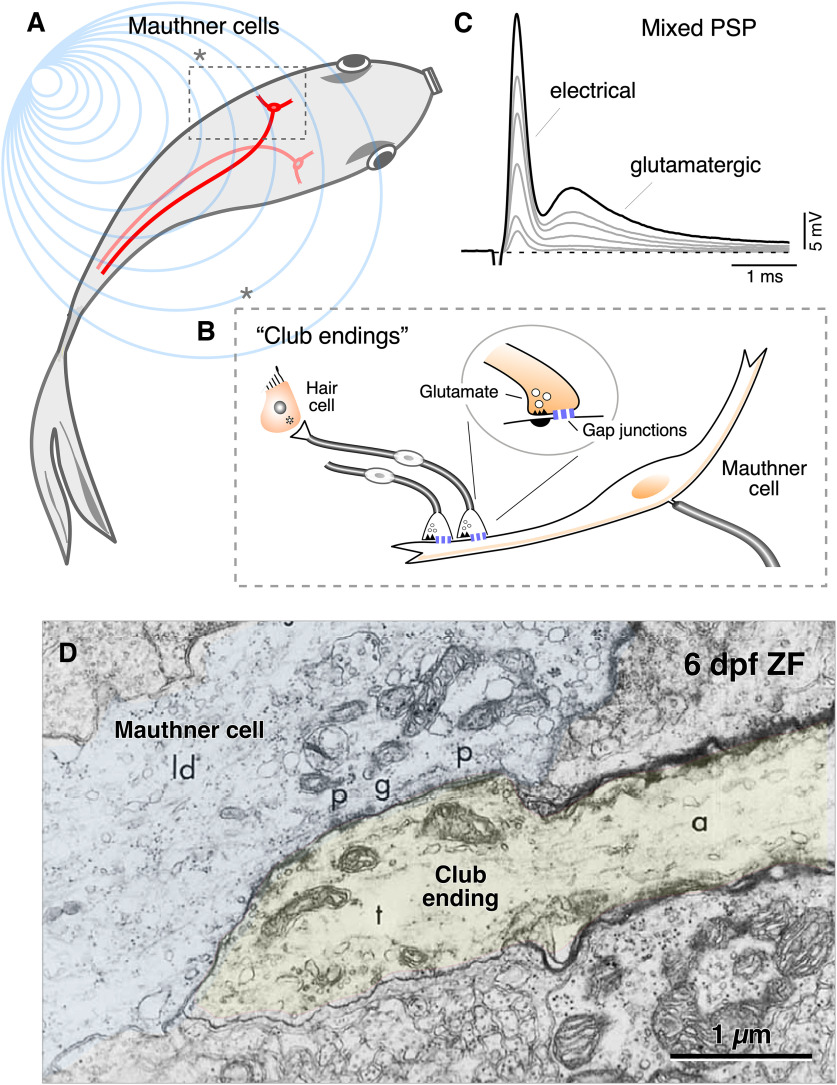
Club endings are identifiable synaptic terminals on the lateral dendrite of the M-cell. ***A***, M-cells initiate auditory-evoked escape responses in fish. ***B***, Saccular auditory afferents terminate as identifiable “mixed,” electrical and chemical, “Large Myelinated Club endings” (Club endings) on the lateral dendrite of the M-cell. ***C***, Stimulation of the posterior branch of the VIIIth nerve where saccular afferents run, evokes a mixed synaptic response composed by an early electrical component that is followed by a delayed, more prolonged, chemically-mediated response. The mixed synaptic response is graded with stimulus intensity, as a result of the activation of different numbers of Club ending afferents. (***A***, modified with permission from Faber and Pereda, 2011; ***B***, ***C***, modified with permission from Pereda et al., 2004.) ***D***, Early electron micrograph shows a putative Club ending (yellow) terminating on the lateral dendrite of the M-cell in a 6-dpf zebrafish. (Modified from Kimmel et al., 1981, with permission.)

**Figure 2.**
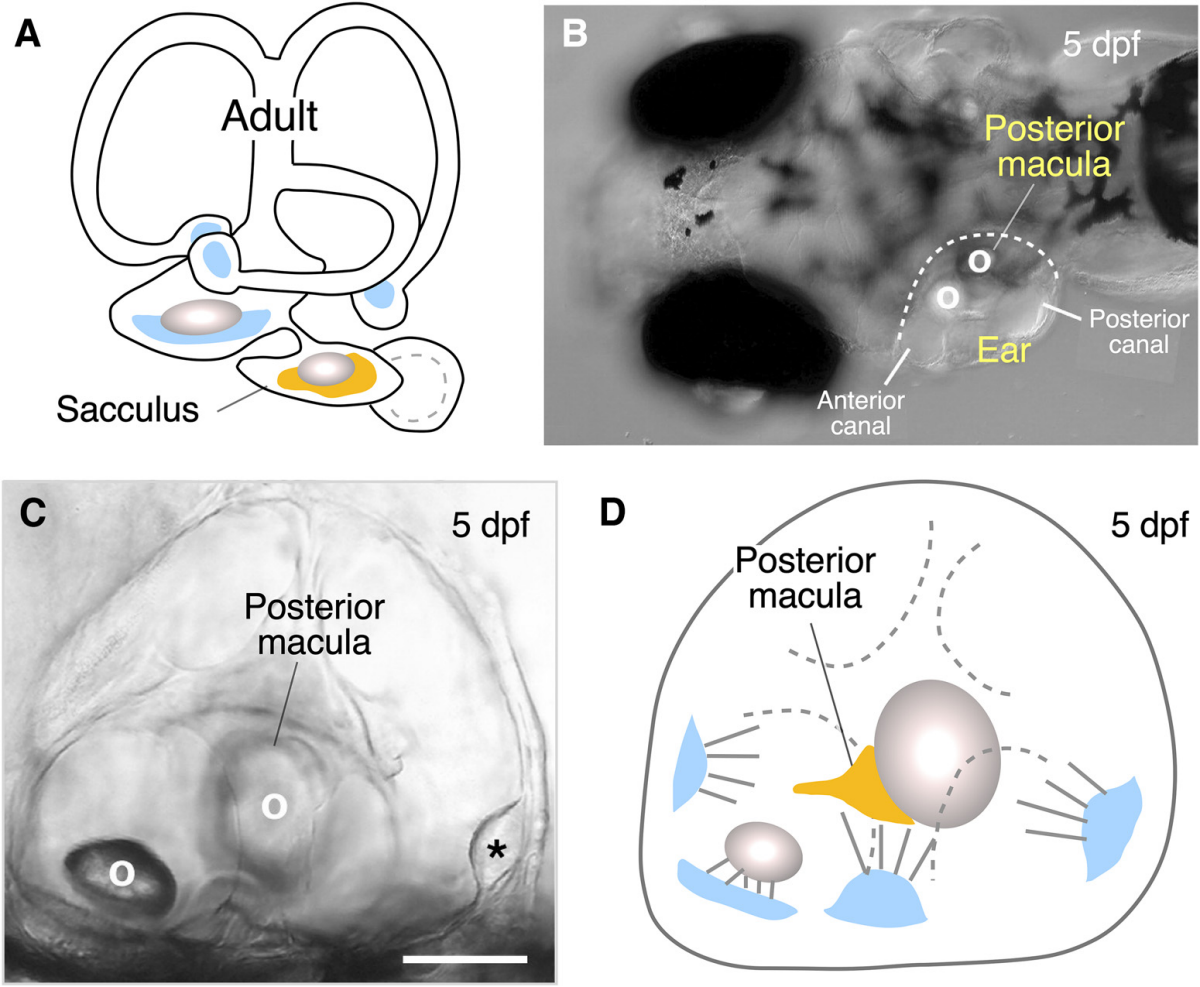
Auditory afferents terminating as Club endings in adult and larval fish M-cells originate from different ear organs. ***A***, The cartoon illustrates the anatomy of the ear of an adult zebrafish. Afferents ending as Club endings in the M-cell contact hair cells in the saccular macula (orange), the main auditory organ in fish. ***B***, Top view of a 5-dpf zebrafish. Each ear has two noticeable otoliths (denoted as O) associated with developing end organs. The posterior otolith is associated to the posterior macula. ***C***, Lateral view of the ear of a 5-dpf larval zebrafish. Calibration bar: 60 μm. The asterisk indicates the posterior canal. ***D***, Cartoon illustrates the different components of the developing ear in larval zebrafish. The posterior macula (orange), associated to the posterior otolith, will give rise to the sacculus in the adult fish. [Cartoons (panels ***A***, ***D***) and images (panels ***B***, ***C***) were redrawn and modified, respectively, with permission from Nicolson, 2005].

Most studies investigating the properties of Club endings were conducted in adult goldfish (Fig. 1*A*,*B*) at which afferents originate in the sacculus (Bartelmez, 1915), the main auditory organ in fish (Fay and Popper, 1974, 2000; Fig. 2*A*). In this fish, the synaptic responses of Club endings can be evoked in the lateral dendrite of the M-cell by stimulating the posterior branch of the VIIIth nerve (Fig. 1*C*), where saccular afferents run (for review, see Cachope et al., 2016). However, the ear of larval zebrafish is formed by transient developing structures that will later give rise to the different adult ear end organs. Consequently, Club ending afferents originate from a different organ than adult goldfish and their axons follow a different trajectory to contact the lateral dendrite of the M-cell. Here, we report detailed methods that allow recording synaptic transmission from Club endings in larval zebrafish by selectively stimulating these auditory afferents at specific structures of the developing ear, thus making it possible to explore their properties in a genetically tractable animal model that is also amenable for live imaging and to correlate them with specific behaviors.

## Materials and Methods

The original description of the Club endings in larval zebrafish was reported previously (Yao et al., 2014). This included information on their origin, number and distribution, molecular composition, and basic functional properties obtained by combining immunolabeling with confocal microscopy and freeze fracture electron microscopy and electrophysiology. Here, together with the detailed procedures on how to reliably obtain synaptic responses during electrophysiological recordings of the M-cell (specifically, why positioning the electrode near the posterior macula is more efficient in stimulating these auditory afferents in isolation) we now include a description of the manipulations that demonstrate the electrical and chemical nature of the components of the mixed synaptic response evoked by stimulation of these afferents (Lasseigne et al., 2021). The materials mentioned during the description of the dissection and recordings are listed in Table 1.

**Table 1:** Materials and chemicals used for dissection and electrophysiological recordings

Item	Manufacturer	Notes
Fluorescent stereomicroscope, for larval dissection	Leica MZ10-F, or equivalent	Equipped with a plan apo 1× objective, fluorescent lamp, and appropriate filters for fluorescence
Upright microscope	Zeiss Axioscope, or equivalent	Equipped with 10×, 20×, and 40× objectives; microscope should be also equipped with DIC/IR optics
Micromanipulator/s	Sutter Instruments MP-285, or equivalent	
Amplifier with A/D converter and recording software connected to computer	Molecular Devices Multiclamp 200 series amplifier, or equivalent	Computer equipped with P-Clamp acquisition and analysis software or equivalent
Stimulator	Digitimer DS3, or equivalent	Constant current isolated stimulator
Borosilicate capillary glass for recordings	World Precision Instruments	Thinwall glass OD/ID 1.5/1.2 mm (catalog #TW150F-3)
Theta glass	World Precision Instruments	For stimulating electrodes OD/ID 1.5/1.02 mm (catalog #TST150-6)
Transfer pipettes	Fisher Scientific	Pipettes to transfer larval zebrafish (catalog #13-678-20A)
Electrode pipette puller	Sutter Instruments P-97, or equivalent	
Fluorodish cell culture dish 35 mm, 10 mm well	World Precision Instruments	Cell culture dish with glass bottom covered with Sylgard (catalog #FD3510-100)
Mini-Petri dishes	Sigma	60-mm diameter (catalog #5481-500EA)
MS-222	Sigma	MS-222 (0.03%) solution buffered with sodium bicarbonate, pH 7.4 (adjusted with NaOH)
Forceps	Fine Science Tools	Dumont #4 Forceps (catalog #11242-40)
Tungsten wire to make custom-made pins	AM Systems	Tungsten wire, size 0.002 inches bare diameter (catalog #795500)
Tungsten wire to make the dissection pin	AM Systems	Tungsten wire, size 0.005 inches bare diameter (catalog #797600)
MA	Sigma	Catalog #6385-02-0
CNQX	Tocris	Catalog #0190
DAP5	Tocris	Catalog #0106
Pneumatic Transducer Tester	Fluke Biomedical	DPM1B
d-tubocurarine	Sigma	10–15 μm d-tubocurarine in external solution (in mm): 134 NaCl, 2.9 KCl, 2.1 CaCl_2_, 1.2 MgCl_2_, 10 HEPES, and 10 glucose, pH adjusted to 7.8 with NaOH

### Procedures for dissection of larval zebrafish

**Figure 3.**
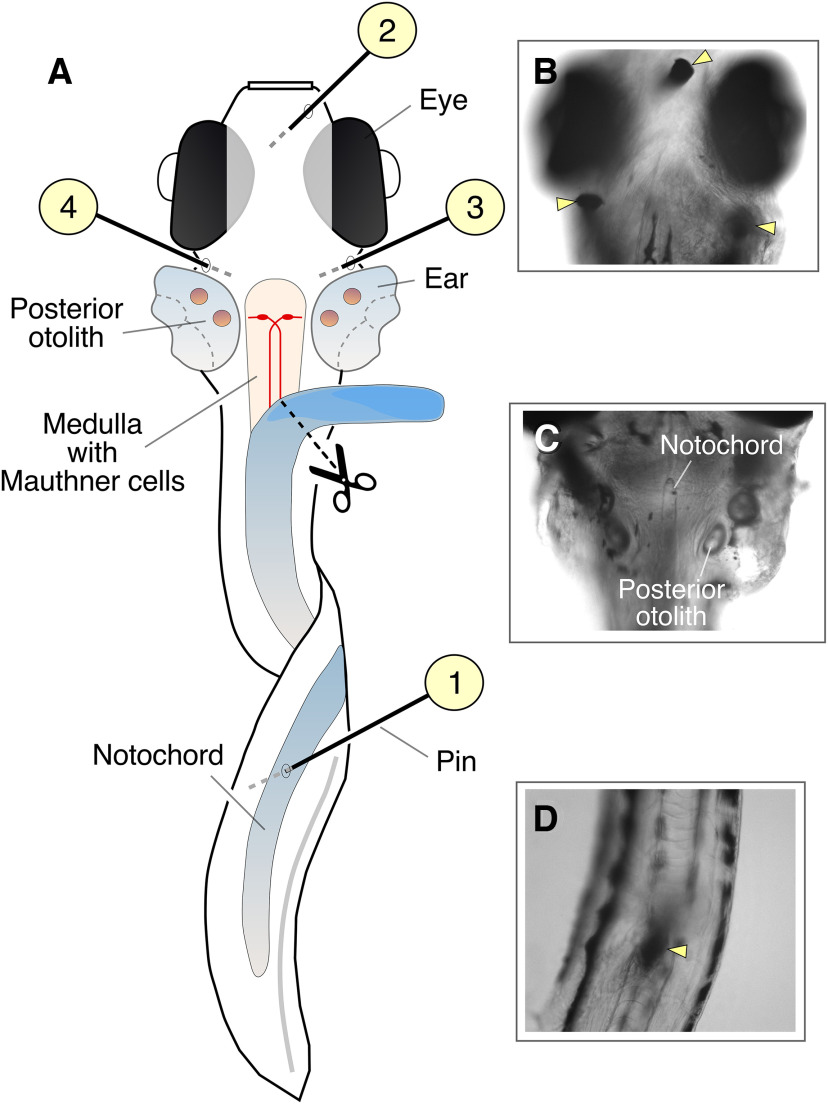
Access to the M-cells using a ventral dissection approach. ***A***, Cartoon illustrates the larva after the removal of the yolk sack, gills, and heart. Numbers indicate the position of the pins used to keep the larva in place and the sequence of their placement. ***B***, Image shows the head of the larva after dissection. Yellow arrowheads indicate the position of the pins. ***C***, Image shows the ventral side of the larva after the gills and heart were removed. Note the medial position of the notochord. ***D***, Image of the tail, held laterally with a pin (yellow arrowhead).

The procedure describes how to immobilize a larval zebrafish to expose the ventral side of the medulla for electrophysiological recordings (the described procedures are in accordance with NIH guidelines for use of Zebrafish in the Intramural Program and approved by the institutional IACUC of the Albert Einstein College of Medicine). In contrast to the more commonly used dorsal approach, the ventral approach (Masino and Fetcho, 2005; Koyama et al., 2011) is particularly convenient as it allows a better visualization of the M-cells, which are located ventrally in the medulla, using differential interference contrast (DIC) optics. Also important, the ventral approach avoids traveling with the electrode through the cerebellum and most of the medulla, thus minimizing its contamination with cellular debris. The ventral method has been previously described and currently used in various laboratories. Briefly, to begin with the dissection, using transfer pipettes, place a few 5–6 d postfertilization (dpf) zebrafish into the MS-222 solution (0.03–0.2%, pH adjusted to 7.4 with NaHCO_3_). Wait for ∼60 s and gently tap on the Petri dish to test for the larvae to be fully anesthetized (when no escape response is observed). Once they stop responding to tapping, rapidly screen fish under dissecting microscope to select larvae that are positively labeled with the fluorescent marker of choice [this step is not necessary for recordings of regular wild-type (WT) larvae]. For the electrophysiological recordings illustrated in this paper, we employed Tol056 transgenic lines, which carry a GFP fluorescent marker under control of the promoter for the Heat Shock Protein 70, and, which include the M-cells (Satou et al., 2009). In this transgenic fish line, GFP-positive (GFP+) fish can be easily screened by looking for GFP expression in the brain and/or retina. Transfer GFP+ (or WT) fish into a minipetri dish containing “external” solution with 10–15 μm d-tubocurarine to paralyze larvae [external solution contains (in mm): 134 NaCl, 2.9 KCl, 2.1 CaCl_2_, 1.2 MgCl_2_, 10 HEPES, and 10 glucose, pH adjusted to 7.8 with NaOH]. Transfer one of the larvae into a cell culture dish with glass bottom (FluoView, WPI), covered with Sylgard (∼150 μl). Place the larva on its back, ventral side-up, using custom-made small pins of ∼25 μm in diameter. These pins can be made from tungsten wire (see Table 1) using electrolytical procedures (Brady, 1965; Bowen, 2010). Place the first pin through the notochord in the tail, approximately at the end of the yolk sack (Fig. 3*A*). Then, carefully, place a second pin in the mouth. Finally, place a pin on each side of the animal, exactly in the space between the eye and the ear (Fig. 3*A*,*B*). Be mindful not to place the pin through the ear, as it will compromise the ability to stimulate the auditory afferents. Placing of the lateral pins also provides the opportunity to align the larva on its back, as symmetrically as possible. This will facilitate the removal of gills, heart, and notochord to expose the ventral side of the medulla and exposing the notochord. To proceed with the removal of these structures, place first one arm of the forceps (Dumont #4) under the gills at the level of the mouth and slowly lift them up. They will start coming off easily, without much effort. Then, grab the gills with the forceps and start pulling caudally, in the direction of the tail. Continue pulling until the gills come off together with the heart, yolk sack and swim bladder (if during this process the gills come apart, use the forceps to remove the remaining pieces of gills, yolk sack, and swim bladder). Avoid breaking the yolk sack, as its contents will decrease visibility during the dissection. If this happens, quickly flush with extracellular solution to remedy the problem. The removal of gills, heart, yolk sack and swim bladder will make possible to visualize the notochord, a denser and more refractive structure that can be clearly identified at the base of the developing cranium (Fig. 3*C*). The most rostral portion of the notochord needs to be removed to finally expose the ventral side of the larva’s medulla. For this purpose, a sharp custom-made dissecting tungsten pin is used to slowly detach the rostral end of the notochord from the rest of the supportive collagenous cranium. To “cut” the cartilage around the rostral tip end of the notochord, use the dissecting pin and apply soft pressure around its edges while moving in the direction of the tail, until it slowly starts detaching itself due of its relative larger rigidity. Once the rostral tip end of the notochord is detached, continue “cutting” the cartilage on each side of the notochord, always moving in the caudal direction. It is critical to cut symmetrically by alternating sides until a significant portion of the notochord has been fully released from the larva (Fig. 3*A*). Cut the detached portion of the notochord using microscissors (Ultra Fine Clipper Scissors II, Fine Science Tools). Transfer the dish with the dissected larva to the microscope in the electrophysiology set up, whose stage should be adapted to hold the circular FluoView dish. The dish containing the dissected animal will be superfused with external solution for the remaining of the experiment to preserve its viability and the application of pharmacological agents.

### Procedures for whole-cell recording and afferent stimulation

**Figure 4.**
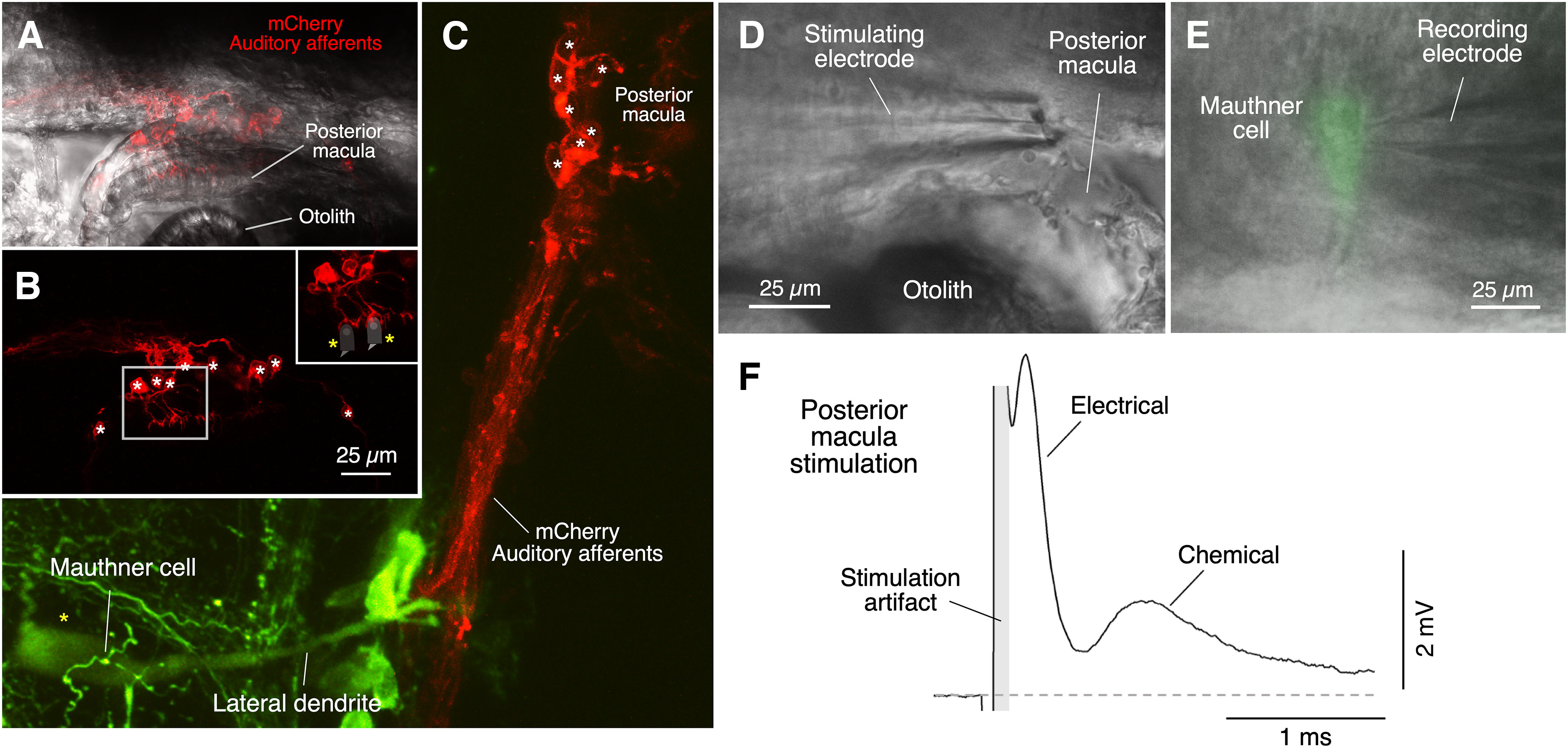
Club endings in larval zebrafish originate from the posterior macula of the developing ear. ***A***, Auditory afferents labeled by expression of mCherry in an auditory afferent Gal4(y256) contact hair cells in the posterior macula which is in close contact with the posterior otolith (5-dpf zebrafish). Confocal image showing mCherry fluorescence overlapped with a DIC image of the area. ***B***, mCherry fluorescence shows cup-shaped calyceal terminations of the dendrites of the auditory afferents on putative hair cells (data not shown; the hair cell is illustrated by a cartoon in the inset). Note the large somata (asterisks) of the afferents clustered in the vicinity of the posterior macula. ***C***, Auditory afferents originated in the posterior macula (red, mCherry) terminate on the lateral dendrite of the M-cell (green, backfilled with rhodamine). Note the large size and clustering of the somata of the auditory afferents in the immediate vicinity of the posterior macula and otolith. ***D***, DIC image of a 6-dpf zebrafish M-cell obtained after exposing the medulla using the ventral dissection approach. The stimulating electrode (bipolar theta glass) is positioned in the posterior macula near the otolith ***E***, Whole-cell recording electrode of the M-cell in a Tol056 zebrafish in which the M-cells express GFP. Images obtained with DIC and fluorescence were superimposed. ***F***, Stimulation of afferents in the vicinity of the posterior macula evokes a mixed synaptic response in the M-cell, composed by a larger and short early response which is followed by a longer lasting smaller response reminiscent of that observed in adult goldfish following stimulation of saccular afferents in the posterior VIIIth nerve. The first component is “riding” the stimulus artifact (indicated in gray) suggesting fast conducting axons and absence of synaptic delay (***E***, ***F*** are modified from Yao et al., 2014, with permission).

Once in the recording stage, orient the FluoView dish so the fish lies horizontally with the head toward the left. This will facilitate the access of the recoding electrode attached to the manipulator at a 22° angle that in our set up is located on the right side of the stage (directions can be reversed if the recording electrode is located on the left side). Proceed to place the stimulating electrode which is also attached at a 22° angle to a second manipulator, in this case on the left side of the microscope stage. In larval zebrafish, the dendritic processes of Club endings contact hair cells located in an end organ known as the “posterior macula” (Fig. 2*B–D*), which serves as auditory organ at this developmental stage. The position of this organ can be easily identified by the presence of the posterior otolith. Otoliths are oval calcareous bodies attached to various sensory macula in the inner ear of fishes, whose relative movement produces deflection of stereocilia in hair cells located at the sensory epithelium. In each ear of 4- to 10-dpf zebrafish, there are two otoliths that can be easily identified as highly refringent oval structures under the microscope (Fig. 2*B*,*C*). As illustrated in Figure 3*D*, the otolith is closely associated to the posterior macula and to the cell bodies of the Club ending afferents (Fig. 4*A–C*). Initially, the stimulation electrode (a theta glass pipette arranged for bipolar stimulation filled with extracellular solution) should be carefully placed on top of the posterior otolith. The final position of the electrode will be adjusted during whole-cell recordings (see below) while monitoring the synaptic responses evoked by electrical stimulation. This allows to optimize the position of the tip of the electrode with respect to the cell bodies of the Club ending afferents (Fig. 4*C*). The final position is generally found to lie rostral to the position of the otolith (Fig. 4*D*). The M-cells can be identified in the dissected larva’s medulla by its larger size either under GFP fluorescence or under DIC in WT animals. The two M-cell somata can be also identified by finding the more easily identifiable large M-cell axons at the spinal cord and then following them from the spinal cord to the hindbrain until their decussation, after where they shortly join the M-cell soma.

For whole-cell recordings, the recording pipette (see Table 1) should have 4–5 MΩ of resistance when filled with the following internal solution (in mm): 105 K-methanesulfonate, 2 MgCl_2_, 2 CaCl_2_, 4 Na_2_ATP, 0.4 Tris-GTP, 10 K_2_-phosphocreatine, 10 HEPES, 10 EGTA, and 25 mannitol, pH adjusted to 7.2 with KOH. With the amplifier in “voltage clamp” mode, enter the bath with the recording pipette using steady positive pressure (∼60 mmHg) by using a Fluke Biomedical DPM1B Pneumatic Transducer Tester attached to the tubing connected to the pipette holder on the head stage. Once the recording pipette is close to the brain, penetrate the brain caudal to the M-cell and immediately decrease the positive pressure to 40 mmHg. The tip of the pipette should be ∼500 μm away from the soma of the M-cell. Keep advancing the pipette diagonally (step size setting of the Sutter MP-285 manipulator: slow, 0.2 μm/click) and at a 22° angle in the direction of the M-cell. Once in front of cell, slowly advance the pipette while simultaneously monitoring the pipette resistance. When the tip of the pipette touches the cell, its resistance will increase. Remove the positive pressure immediately and start applying negative pressure (−10 to −20 mmHg). Set a negative holding potential (−60 mV) and wait for seal formation (increase negative pressure by −5 mmHg if necessary). Once a gigaohm seal is formed (at least 1 GΩ), break-in by using a rapid increase in negative pressure with a 1 ml syringe or simply by mouth (if safety protocols allow). Once the whole-cell recording is established, switch the amplifier to the “current clamp” mode. In healthy cells, the resting membrane potential should range between −60 and −70 mV. Using a subthreshold depolarizing pulse, adjust the electrode resistance (bridge) either manually or by using the automated function if available in your amplifier. Evoking an action potential in the M-cell usually requires a pulse of 3–4 nA, given the relative much lower input resistance of this cell (Ali et al., 2000; Koyama et al., 2011; Yao et al., 2014).

Once the intracellular recording is stable, while monitoring the amplitude of the evoked response, move the stimulating electrode within a few microns around to optimize its position relative to cell bodies of the Club ending afferents. The optimal position corresponds to that evoking the largest synaptic response amplitude for the same stimulation strength. Further increase of the intensity of stimulation will result in an increase of the mixed synaptic response, characteristic of Club endings, as a result of the activation of a larger number of these afferents (Figs. 1*C*, 4*F*). If necessary, the stimulus electrode can be carefully repositioned to optimize the amplitude of the mixed synaptic potential and avoid the potential contamination produced by the unintended stimulation of other afferent inputs to the M-cell. A minimal amount of current is required for evoking the synaptic response when the electrode is ideally positioned near the cell bodies of the Club endings, in the vicinity of the posterior macula. Maximal activation of Club endings is achieved when the amplitude of the electrical component does not further increase with increased stimulation; beyond this maximal amplitude increased stimulation results in the presence of additional synaptic potentials with longer latencies as a result of the activation of additional afferent inputs to the M-cell with higher threshold for extracellular stimulation.

## Experimental Recordings

Because of the availability of the fish and easy experimental access, goldfish M-cells (Fig. 1*A*, depicted in red) and Club endings have traditionally served as a valuable experimental model to study mechanisms underlying synaptic transmission (Pereda et al., 2004; Korn and Faber, 2005). Auditory afferents terminating as “Club endings” on the lateral dendrite of the M-cell originate in the sacculus (Fig. 2*A*). Because these large terminals combine GJs with specializations of chemical transmission (Robertson et al., 1963; Kohno and Noguchi, 1986), the stimulation of the posterior branch of the VIIIth nerve (where these saccular afferents run) evokes a mixed synaptic response composed of an early electrical component mediated by the GJs and a delayed, longer-lasting, synaptic response mediated by glutamate (Fig. 1*C*; Furshpan, 1964; Lin and Faber, 1988a; Wolszon et al., 1997). The two components can be easily identified because of the unusually fast time constant of the M-cell (Fukami et al., 1965). The emergence of zebrafish as animal model has provided the opportunity of investigating the functional properties of Club endings combining electrophysiology with additional experimental approaches. The M-cell and its associated network is already formed and functional at 4 dpf (Takahashi et al., 2002; Koyama et al., 2016), when the development of the swim bladder provides buoyancy, thus allowing swimming. Early evidence of the presence of these terminals in larval zebrafish was obtained with electron microscopy (Kimmel et al., 1981; Fig. 1*D*) and were more recently functionally and molecularly characterized (Tanimoto et al., 2009; Yao et al., 2014; Miller et al., 2017a; Lasseigne et al., 2021).

While low-intensity stimulation on the posterior branch of the VIIIth nerve allow selective stimulation of Club ending afferents, which is facilitated by the relative larger diameter of these afferents (for review, see Cachope et al., 2016), selective stimulation is more difficult to achieve in zebrafish larvae. The main reason behind this difficulty is that Club endings in larval zebrafish arise from a different auditory organ, the posterior macula, which will later give rise to the sacculus among other end organs (Nicolson, 2005; Fig. 2*B–D*). The position of the anterior and posterior macula can be recognized by presence of their respective otoliths (Fig. 2*B*,*C*). The posterior macula contains the hair cells synapsing on dendritic processes of the auditory afferents that terminate as Club endings on the M-cell. The different organs of the developing ear and their relative position is illustrated in Figure 2*D* (note the differences with the cartoon in Fig. 2*A*, representing the different end organs in an adult fish).

The use of the auditory afferent Gal4(y256) (Marquart et al., 2015) crossed with a UAS:ChR2(H134R):mCherry allowed the visualization of the trajectory of the Club ending afferents to the lateral dendrite of the M-cell. The auditory afferent Gal4 line expresses transgenes under UAS control strongly in auditory afferents by 6 dpf, with some scattered additional neurons in the brain. The approach revealed that the cell bodies of the afferents are relatively large (∼7–8 μm) and clustered together in the immediate vicinity of the posterior macula (Fig. 4*A–C*). The full trajectory from the posterior macula to the lateral dendrite of the M-cell can be appreciated in Figure 4*C*, in which the M-cell and other spinal projecting neurons were retrogradely labeled by application of dextran (70,000 MW) tetramethylrhodamine in the spinal cord and imaged with the help of a confocal microscope (Fig. 4*C*).

The anatomic arrangement of the cell bodies, clustered together in close proximity of the posterior macula, and their relative larger size (compare the diameter of the cell bodies with that of the axons and dendritic processes) provides with the opportunity of selectively activating the auditory afferents with an extracellular electrode using low stimulation intensity, as larger cellular processes have lower threshold for extracellular stimulation (Dodge, 1979; Moore and Westerfield, 1983). An advantage of larval zebrafish is the small size and transparency of its brain. This allows for visualization of the M-cells, which are around 90 μm deep inside the hindbrain in 4- to 6-dpf zebrafish, using an upright microscope with a DIC objective and an infrared filter after exposing the medulla with the ventral dissection approach (See procedures for dissection section). During whole-cell recordings of the M-cell (Fig. 4*E*), bipolar extracellular stimulation of these afferents using a theta glass pipette positioned near the posterior macula (Fig. 4*D*) evokes a synaptic response on the M-cell (Fig. 4*F*), which consists of a fast early component followed by a smaller longer-lasting component that is reminiscent of the mixed synaptic potential evoked by stimulation of Club endings in adult goldfish (Fig. 1*C*).

### Validation of the electrical and chemical nature of the mixed synaptic response

**Figure 5.**
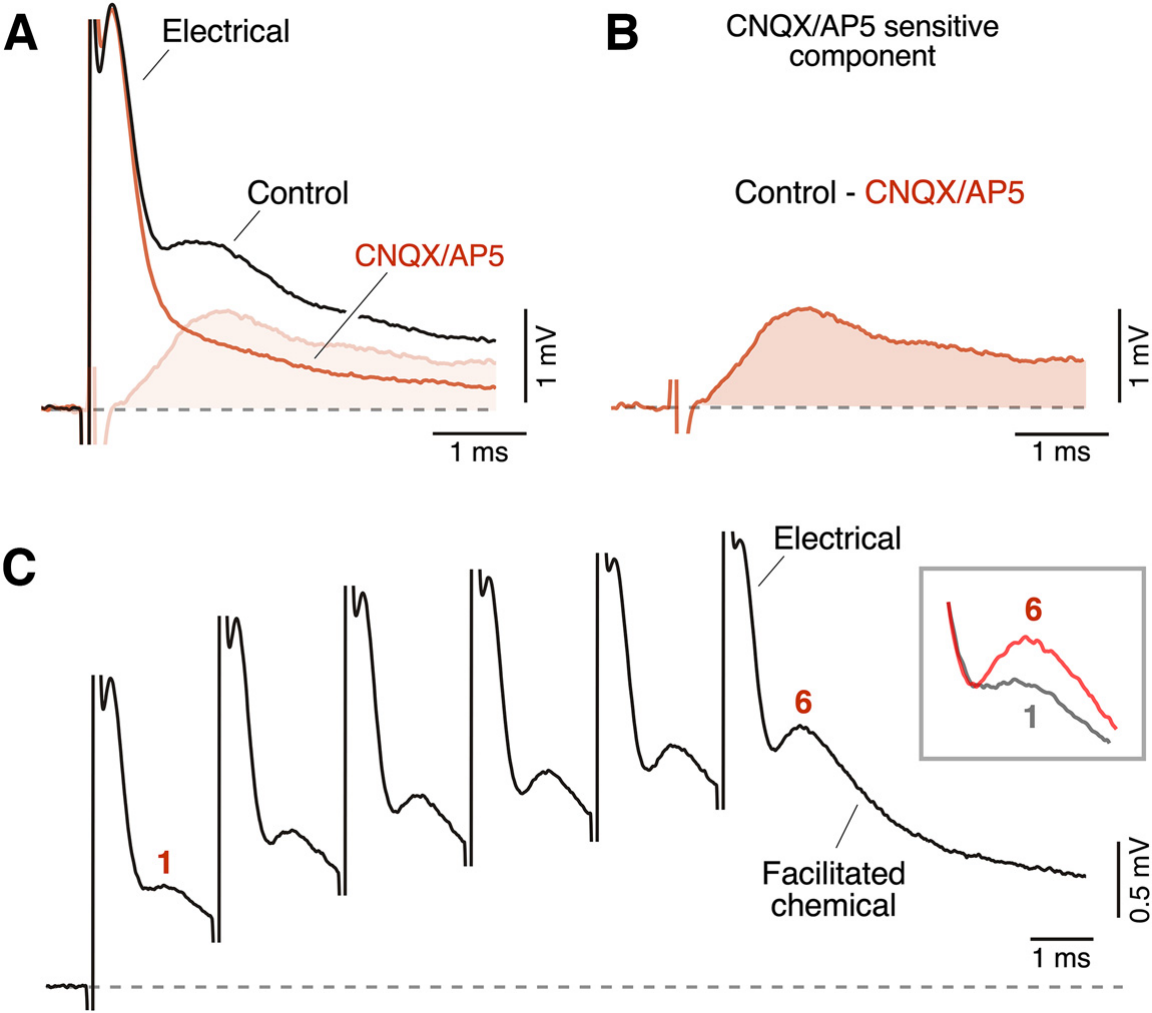
The delayed component of the mixed synaptic response is mediated by glutamate. ***A***, Application of a combination of antagonists of glutamate receptors (CNQX and DAP5, 20 μm each) suppresses the delayed, longer-lasting, component of the synaptic response without affecting its early component. The subtraction of the recording trace after drug application (red) from that obtained in control (black) reveals the time course of the glutamatergic component. ***B***, The subtracted response is illustrated at higher amplitude. Large dendritic synaptic potentials in the M-cell do not readily return to baseline, likely because of the activation of a voltage-dependent dendritic conductance (Szabo et al., 2006). Recordings in ***A***, ***B*** are selected unpublished examples from a previously published dataset (Lasseigne et al., 2021). ***C***, Application of multiple pulses with a 2-ms interval (500 Hz) leads to facilitation of the chemical component. Inset, The amplitude of the chemical components after the first (1) and last (6) stimulation pulse are illustrated superimposed. Each trace represents the average of 10 selected individual recordings.

In goldfish, synaptic transmission is mediated by co-existing GJs containing channels formed by two homologs of Cx36 and by the release of glutamate from presynaptic release sites. Thus, while the early component of the synaptic response represents the coupling of the action potentials occurring at active presynaptic terminals, the late and longer-lasting component represents the release of glutamate produced by the increase in Ca^2+^ evoked by these action potentials (Wolszon et al., 1997). Glutamate also mediates chemical transmission in larval zebrafish Club endings (Lasseigne et al., 2021). Application of CNQX and AP5, antagonists of AMPA and NMDA glutamate receptors, respectively, blocked the late longer-lasting component of the synaptic response without affecting the early component (Fig. 5*A*). Subtraction of traces obtained in the presence and absence of glutamate receptor antagonists illustrates the amplitude and time course of the CNQX and AP5 sensitive component (Fig. 5*B*). A characteristic feature of glutamatergic synapses at Club endings is their ability to undergo synaptic facilitation in response to multiple stimulation pulses (Pereda and Faber, 1996; Wolszon et al., 1997; Curti et al., 2008). Stimulation with a burst of six pulses at 2-ms interval (500 Hz) showed a progressive facilitation of the late component, characteristic of the synaptic facilitation observed at goldfish Club endings (Fig. 5*C*; Pereda and Faber, 1996). Combined with its pharmacological sensitivity, these release properties indicate that, as in adult goldfish, the late component of the mixed synaptic response in larval zebrafish is also chemically-mediated.

**Figure 6.**
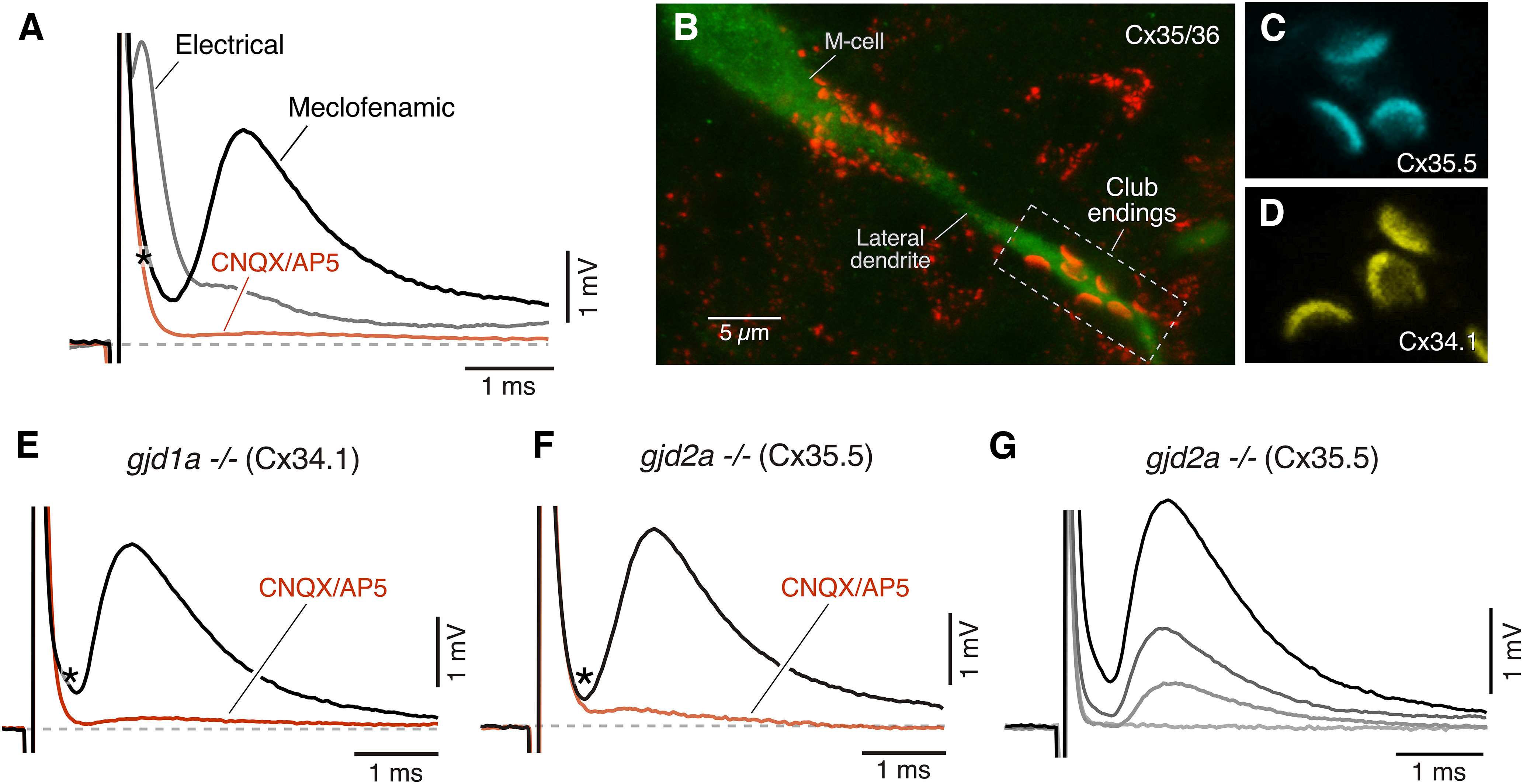
The first component of the mixed synaptic response requires functional GJ channels. ***A***, Superimposed synaptic responses obtained in a 5-dpf zebrafish before (black trace) and after (red trace) adding MA (meclofenamic, 200 μm) to the perfusion solution. The remaining synaptic response was blocked (black trace) by adding CNQX/DAP5 (20 μm each) to the perfusion solution. Asterisk (*) here and in panels ***E***, ***F*** indicates the time at which the electrical component was expected if present. ***B***, Confocal image of a cell stained with anti-GFP (green) and anti Cx35/36 (red). The contact areas of individual Club endings are easily identifiable on the distal portion of the lateral dendrite of the M-cell because of the larger size and characteristic segregation. ***C***, ***D***, GJ channels at Club endings are formed by heterotypic channels made of presynaptic Cx35.5 (*gjd2a/Cx35.5^–/–^*) and postsynaptic Cx34.1 (*gjd1a/Cx34.1^–/–^*). Images represent Club ending contact areas illustrated at higher magnification stained with either anti-zebrafish-Cx35.5 (***C***, cyan) or anti-zebrafish-Cx34.1 (***D***, yellow). ***E-F****, gjd1a/Cx34.1^–/–^* and *gjd2a/Cx35.5^–/–^* mutant zebrafish have no detectable electrical component (black traces). The remaining synaptic response was blocked by bath application of CNQX and DAP5 (20 μm each). Images and recordings in panels ***A*–*D***, ***F*** are unpublished examples from a previously published dataset (Lasseigne et al., 2021). ***G***, Synaptic recording obtained in a *gjd2a/Cx35.5^–/–^* fish using different stimulation intensities. No electrical component was observed even at higher stimulation intensities. Each trace represents the average of at least 10 individual recordings.

The electrical nature of the remaining CNQX/AP5 nonsensitive component was recently established by a combination of pharmacological and genetic approaches (Lasseigne et al., 2021). As illustrated in Figure 6*A*, application of the GJ blocking agent meclofenamic acid (MA; black trace) blocks the early component of the mixed synaptic response, which in turn causes a parallel increase in the late component. This increase is in part because of an increase in the input resistance of the M-cell as result of the elimination of the current leak facilitated by GJs toward coupled cells (Lasseigne et al., 2021). As expected, the meclofenamic residual component was blocked by subsequent addition of CNQX and AP5 to the bath solution (Fig. 6*A*). Finally, GJ channels at Club endings are formed by two homologs of the mammalian Cx36, a widespread neuronal connexin, arranged in a heterotypic fashion. While the presynaptic hemichannel is formed by Cx35.5 encoded by the gene *gjd2a*, the postsynaptic hemichannel is formed by Cx34.1 and encoded by the gene *gjd1a*. Anatomically the contact areas of the Club endings can be easily identified with immunolabeling by their larger size (∼2 μm in diameter) in the distal portion of the lateral dendrite (Fig. 6*B*) with an antibody that recognizes both Cx35.5 and Cx34.1. The presence of these connexins at these contact areas can be exposed using specific antibodies against each of these proteins (Fig. 6*C*,*D*). Mutations of the genes encoding Cx35.5 and Cx34.1 leads to the disappearance of the early component of the mixed synaptic response (Lasseigne et al., 2021). Whole-cell recordings of M-cells in *gjd1a^–/–^* and *gjd2a^–/–^* zebrafish showed an absence of the early component of the mixed synaptic response, as usually obtained in response to stimulation of auditory afferents in the ear (Fig. 6*E*,*F*), confirming it represents an electrical component mediated by GJ channels. The stimulation of afferents at the ear with pulses of increasing strength exposed the total absence of electrical transmission in these mutants (Fig. 6*G*), as no electrical component was detected regardless of stimulation strength.

## Conclusions

Because of their experimental accessibility, identifiable large synaptic terminals on the M-cell known as Large Myelinated Club endings have provided an useful window to advance our understandings of the mechanisms underlying chemical and electrical transmission in the vertebrate brain (Bartelmez and Hoerr, 1933; Bodian, 1937; Robertson et al., 1963; Furshpan, 1964; Pereda et al., 2004). The main experimental advantage of these terminals lies on their unique identifiability for both anatomic and physiological experimentation as result of their larger size and location on a uniquely experimentally accessible postsynaptic cell. Such experimental access has more easily allowed correlations of structure and function to expose various structural and molecular determinants of electrical synaptic transmission (Robertson et al., 1963; Pereda et al., 2003; Rash et al., 2013; Miller et al., 2017b; Lasseigne et al., 2021).

The methods described here will make possible to record synaptic transmission from these auditory synapses in larval zebrafish, an animal model that offers unparalleled advantages to explore the cellular determinants of neural circuits in the vertebrate brain (Burgess et al., 2009; Portugues and Engert, 2009; Ahrens et al., 2012; Dunn et al., 2016; Koyama et al., 2016; Jain et al., 2018), as well as detailed molecular determinants underlying synaptic transmission (Wen et al., 2013, 2016a, b; Brehm and Wen, 2019). The main experimental advantage of the described approach is the identification of a site in the developing ear that allows selective activation of these afferents using minimal current strength, thus allowing recording the synaptic potentials they evoke in the M-cell in isolation. The properties of these terminals are indistinguishable from those of adult goldfish and the chemical and electrical nature of the two components of their mixed synaptic response has been unambiguously established with pharmacological and genetic approaches (Yao et al., 2014; Miller et al., 2017a; Lasseigne et al., 2021). These findings are consistent with the fact that the M-cell network is already anatomically and functionally established at early larval stages (Takahashi et al., 2002; Tanimoto et al., 2009; Koyama et al., 2011) and therefore capable of participating in behavioral responses.

Historically, Club endings have greatly contributed, since their identification, to advancing our understanding of synaptic structure and mechanisms. The amenability of zebrafish for genetic manipulation and live imaging has the potential of bringing the unique experimental accessibility of these terminals to exciting new levels of discovery.

## Synthesis

Reviewing Editor: Nathalie Rouach, College de France Decisions are customarily a result of the Reviewing Editor and the peer reviewers coming together and discussing their recommendations until a consensus is reached. When revisions are invited, a fact-based synthesis statement explaining their decision and outlining what is needed to prepare a revision will be listed below. The following reviewer(s) agreed to reveal their identity: Linda Dansereau.

This paper aims to introduce the use of larval zebrafish to study Mauthner cells as a model system for studying mixed chemical and electrical synapses. This would expand the usefulness of a genetically modifiable model system to study the role of mixed synapses during development to supplement adult goldfish studies. Mixed synapses are relatively common in lower vertebrates but not so much in mammals. Study of this synapse could provide an opportunity in investigate how electric and chemical synapses coordinate with each other during development or in the context of synaptic plasticity. This coordination may be universal albeit between separated electric and chemical synapses in mammalian brains. It may not be obvious at the moment, but this model system may rise in its application as new neurobiological questions emerge. This manuscript describes detailed procedures for electrophysiological analysis from a mixed synapse in zebrafish auditory system. The authors successfully patched GFP labelled Mauthner cells in the larval zebrafish, stimulated them via the auditory afferents and confirmed they acted as expected via chemical and genetic methods. The paper provides appropriate details as a method paper. Providing a short video of the dissection procedure would be useful and could significantly improve the paper.

While being able to perform this kind of study in zebrafish is an exciting advance, there is no shortage of model systems where synaptic transmission, electric or chemical, can be studied with readily available genetic tools. To increase the impact of this manuscript, the authors should try to convince readers that club ending synapses can solve some unique and significant neurobiological questions that few other model systems can. To do so, the authors should add a paragraph more specifically outlining potential biological questions (developmental, auditory processing, pre- & post-synaptic communications via gap junctions as opposed to signaling molecules) this model systems can uniquely solve.

Major comments

1. Although the authors provide a clear protocol, they could better emphasize how this is different from previously reported methods. There is an unreferenced statement that the ventral method has previously been described although the authors do state that the main contribution is being able to stimulate the auditory afferents to study the circuit in a viable context. However, data in Figure 3E and F is presented as experimental results in the text while the figure legend states it is modified with permission from a 2015/2014 paper. This suggests the method, including stimulating the auditory afferents, has been previously implemented. Some clarification on this is requested to make very clear what is new and improved in this new paper compared to the method published in Yao C et al (2014) Electrical synaptic transmission in developing zebrafish: properties and molecular composition of gap junctions at a central auditory synapse. J Neurophysiol 112:2102-2113.

2. It would be useful to include a diagram of the zebrafish in the ventral orientation to help orient the reader through the procedure. Also, images of the preparation, such as the positioning of the pins, could be included.

3. There are departures from technical writing conventions, including referring to figures out of the order they are labelled and inconsistent use of Fig. or Figure, that warrants another round of proof-reading.

4. Experimental data in Figure 3E and F are modified with permission from a 2015/2014 paper. More recent data should be included instead.

Minor comments:

1. Part of the procedure suggests creating the patch-clamp seal “by using a rapid increase in negative pressure with a 1 ml syringe or simply by mouth”. This suggestion may not pass all institutional safety protocols so recommend including “if safety protocols allow” to the sentence.

2. Please include details on how the images of the 5 dpf zebrafish in figure 2 were obtained.

p.11 2nd para, line 5 near end: will give later give rise...; delete give later

p.12 1st para line 3 & 5: should be Figure 3 not 4

Fig.3 legend: putative hair cell should be marked, by outline or asterisk.

Fig.2C: there is an “*” in the figure but it is not mentioned in the figure legend.
